# Growth performance and emergence of invasive alien Rumex confertus in different soil types

**DOI:** 10.1038/s41598-019-56068-9

**Published:** 2019-12-23

**Authors:** Jeremi Kołodziejek

**Affiliations:** Department of Geobotany and Plant Ecology, Faculty of Biology and Environmental Protection, University of Lodz, 12/16 Banacha St., 90-237 Lodz, Poland

**Keywords:** Ecology, Plant sciences

## Abstract

*Rumex confertus* is known to be one of the most serious invasive weed species infesting meadows, pastures and ruderal lands throughout the Central Europe. *Rumex confertus* was grown in pot experiments using 8 soil types at 5 concentrations of nitrogen (N) and phosphorus (P). Based on harvest data, the variables, seedlings emergence, root: shoot (R: S) ratio, N and P concentration, chlorophyll content, Relative Colimitation Index (RCI) and dry matter allocation to plant components, were determined. N and P addition stimulated the growth of plants in different soils, as reflected by a significant increase in seedling growth parameters such as total plant biomass and shoot biomass. Across all soil types, emergence of seedlings was negatively affected by very high N, but positively affected by increased P availability. This study indicates that Dystric Arenosol, Entic Podzol, Brunic Arenosol and Calcaric Leptosol are unfavorable for *R. confertus* growth, excluding  *R. confertus* completely. Moreover, evidence suggests that plant growth is limited by both N and P, therefore *R. confertus* could be controlled by reducing available N and P content in the soil.

## Introduction

Invasion of exotic plant species is a global phenomenon that poses a critical problem for the conservation and management of many ecosystems^1,2^. Plant invasion is influenced by various environmental factors including both abiotic and biotic factors. Soil type is one of the essential abiotic factors which might affect plants growth. It has long been recognized that that invasive plant species have a wide environmental tolerance that allows them to grow and development on different types of soil, enabling them to easily invade new habitats^3^.

In many terrestrial ecosystems, nitrogen (N) and phosphorus (P) are usually considered the two most common and primary nutrient for plant growth at the biochemical level and are therefore known to play a crucial role in plant growth, especially in invasive plants^4–8^. N is the nutrient element that plays a crucial role in photosynthesis, plant production, protein synthesis and all enzymatic activity, whereas P is involved in photosynthesis, the energy transfer within cells, the transport of carbohydrates and, together with N, it is an important structural element in DNA and RNA^9–12^.

Empirical data shown that both N and P, are more apt to be deficient in natural soils than the others^13^. As N and P availability increases due to agricultural runoff, ecosystems can become more vulnerable to invasion as some invasive plants respond more positively than natives to N- and P-enrichment^14^. For example, Thorpe *et al*.^15^ found that phosphorus concentration in the invasive *Centaurea maculosa* was more than twice that of native species and phosphorus uptake by this invasive species was six times greater than that by the native, *Lupinus argenteus*. Which of the main nutrients limits the growth, development and eventually settling of new habitats depends on the chemical composition of the soil and the availability of nutrients and on the balance between their inputs and outputs^16^.

Several studies have revealed a strong interaction between these two mineral nutrients in plant growth^17–19^. For example, positive interaction between N and P which leads to increase in P absorption have reported for *Eucalyptus grandis* plants^20^. In many terrestrial ecosystems soil pH, soil texture and soil aeration are thought to be the key factors affecting nutrients interaction^21–23^.

*Rumex confertus*, native to Eastern Europe and Asia, is known to be one of the most serious invasive weed species in Central Europe, but its preferred soil types are not documented. It is also necessary to know the individual (N or P) and combined (N + P) effects on seedling emergence, or subsequent growth of *R. confertus*. In particular I addressed the following questions: (1) how emergence of seedlings as well as the growth of shoots and roots of this species are affected by different soil types and N and/or P supply, (2) why *R. confertus* does not invade some soil types, and (3) whether fertilization with P may increase N uptake.

Specific hypotheses were as follows:

1. Biomass allocation across the N and P addition gradients is greater to shoots and lower to roots.
2. Fertilization with P increases growth of *R. confertus* plants and improves leaf nutrient status more than N-fertilization.
3. *Rumex confertus* may be both N and P limited (co-limited), that is, both N and P must be added to obtain a significant increase, because it grows in a wide range of habitats.
4. The addition of N and P enhances leaf chlorophyll content.

## Results

### Comparison in physicochemical properties among different types of cultivation soil

As shown in Table 1, physicochemical properties were different in different soil types. The contents of C, N, K, P were highest in Fluvic Cambisol, they were 6, 9, 4 and 3 times higher, respectively, then those in Dystric Arenosol, which was the poorest soil. Generally all soils were poor in extractable P. Taking pH-KCl value into account, the studied soils ranged from acidic (Dystric Arenosol – pH 5.5) to alkaline (Leptosol – pH 7.3). Soil C levels varied from 0.89% (Dystric Arenosol) to 5.15% (Fluvic Cambisol), and total soil N levels varied from 0.06% (Dystric Arenosol) to 0.56% (Fluvic Cambisol). The Dystric Gleysols soil had the lowest and the Entic Podzol had the highest C: N ratio. There was no distinct physical and chemical difference between Dystric Arenosol and Entic Podzol. Both contained less nutrients than the other soil types and had lower pH.

**Table 1:** Physicochemical properties of the soil (organic matter content, pH and macronutrient content [N, P, K]) used in the pot experiments.

Soil types	C (%)	N (%)	C: N	K (mg 100 g)	P (mg 100 g)	pH-H_2_O	pH-KCL	Soil particle size (%, mm)			
								>2.0	2.0–0.05	0.05–0.002	<0.002
Dystric Arenosol	0.89	0.06	14.8	3.4	2.3	4.2	3.5	3	94	0	0
Entic Podzol	1.25	0.08	15.6	3.6	2.5	4.8	4.2	0.0	96.6	3.4	0.0
Brunic Arenosol	1.9	0.18	10.6	4.8	2.8	4.9	3.4	14.3	56.7	29.0	20.0
Leptosol	2.78	0.21	13.2	4.6	3.5	7.7	7.3	0.5	69.0	25.0	5.5
Fibric Histosol	4.16	0.38	10.9	8.5	3.9	5.0	4.6	3	84	12	2
Fluvic Cambisol	5.15	0.56	9.2	13.3	8.3	6.9	6.6	0.0	23	59	8
Dystric Gleysol	2.82	0.29	9.7	6.4	3.7	5.8	5.3	0.0	14	50	36
Eutric Cambisol	3.67	0.46	8.0	14.7	6.8	6.9	6.0	0.7	53.1	26.2	20

### The influences of four types of soil on the growth – visual observations

In this experiment I selected four representative soil types with quite different textures and physicochemical properties, i.e. Dystric Arenosol (loose texture, the low pH level), Entic Podzol (loose texture, the low pH level), Brunic Arenosol (moderate texture, the low pH level) and Leptosol (dense texture and rich in calcium carbonate). The four soil types used in this experiment had different physicochemical properties. The results of this experiment indicated that during 10 weeks seedlings died shortly after they started growing or else showed poor growth in four soils. The data were not treated statistically as only few plants survived in each soil culture. The seedlings were not measured for aboveground biomass and belowground biomass because of the poor growth conditions and lack of new leaves. Four seedlings which survived on Brunic Arenosol remained at the seedling stage throughout the experiment. During the course of experiment the plants assumed a pale greenish hue. The seedlings grown on Dystric Arenosol and Entic Podzol showed typical N deficiency symptoms; they were small with pale, chlorotic foliage and showed visible darkening of the foliage and pronounced purple discoloration. Typical signs of P deficiency were also found, including increased R: S ratio and reduced biomass.

### Effects of soil type and nutrients on total dry weight, shoot and root biomass and root to shoot biomass (R: S) ratio

Significant (*P* < 0.05) differences were observed in the growth of *R. confertus* plants on the soil used in terms of plant biomass (Table 2). In general, the control non-fertilized plants grew better in the Fluvic Cambisol than in the other three types of soil i.e., Eutric Cambisols, Fibric Histosol and Dystric Gleysols. The total biomass of plants grown in Fluvic Cambisol was about 19–46% greater than of those grown in Fibric Histosol or Dystric Gleysols. Not only the total biomass but also the shoot biomass varied depending on the soil types. It was the largest on Fluvic Cambisol and the smallest on Dystric Gleysol. The R: S tended to be lower in Fluvic Cambisol than in the other three soils.

**Table 2:** Mean values and their standard deviations for total dry weight (DW), shoot and root biomass, root to shoot (leaf + stem DW) (R: S) ratio of *R. confertus* plants grown with and without fertilization in four different natural soil types.

Soil types	Nutrients added	Total dry weight (g DW plant^−1^)	Shoot biomass (g DW plant^−1^)	Root biomass (g DW plant^−1^)	Root: shoot (R: S) ratio
Fluvic Cambisol	C	38.2 ± 0.8^c^	21.3 ± 0.9 ^f^	16.9 ± 0.3^a^	0.79^a^
	N1	39.9 ± 0.5^b^	23.3 ± 0.6^e^	16.6 ± 0.4^a^	0.71^b^
	N2	40.3 ± 0.3^b^	24.3 ± 0.8^d^	15.8 ± 0.4^b^	0.65^c^
	P1	40.5 ± 0.5^b^	25.5 ± 0.6^c^	15.0 ± 0.6^b^	0.59^d^
	P2	40.9 ± 0.5^b^	27.2 ± 0.7^b^	13.7 ± 0.4^c^	0.50^e^
	N1 + P1	41.9 ± 0.9^a^	28.0 ± 0.4^b^	13.2 ± 0.5 ^cd^	0.47^e^
	N2 + P2	42.5 ± 1.1^a^	29.8 ± 0.6^a^	12.7 ± 0.4^d^	0.43^e^
Eutric Cambisol	C	34.8 ± 0.5^c^	18.2 ± 1.0 ^g^	16.6 ± 0.5^a^	0.91^a^
	N1	37.2 ± 0.4^b^	22.1 ± 0.6 ^f^	15.1 ± 0.5^b^	0.68^b^
	N2	37.5 ± 0.6^b^	24.1 ± 0.3^e^	13.4 ± 0.3^c^	0.56^c^
	P1	39.0 ± 0.5^a^	25.6 ± 0.6^d^	13.4 ± 0.6^c^	0.52^c^
	P2	39.1 ± 0.5^a^	27.0 ± 0.8^c^	12.1 ± 0.3^d^	0.45^d^
	N1 + P1	39.5 ± 0.4^a^	28.5 ± 0.4^b^	11.0 ± 0.5^e^	0.39^d^
	N2 + P2	39.9 ± 0.7^a^	29.4 ± 0.6^a^	10.5 ± 0.2^e^	0.36^d^
Fibric Histosol	C	32.5 ± 0.5^c^	17.3 ± 0.8^e^	15.2 ± 0.4^a^	0.88^a^
	N1	35.8 ± 0.4^b^	22.1 ± 0.5^d^	13.7 ± 0.3^b^	0.62^b^
	N2	36.1 ± 0.5^b^	23.7 ± 0.3^c^	12.4 ± 0.2^c^	0.52^c^
	P1	36.3 ± 0.7^b^	24.0 ± 0.7^c^	12.3 ± 0.5^c^	0.51^c^
	P2	36.6 ± 0.6^b^	24.9 ± 0.8^c^	11.7 ± 0.6^c^	0.47^c^
	N1 + P1	37.8 ± 0.5^a^	26.6 ± 0.4^b^	11.2 ± 0.5 ^cd^	0.42^c^
	N2 + P2	38.2 ± 0.6^a^	28.5 ± 0.6^a^	9.7 ± 0.4^d^	0.34^d^
Dystric Gleysols	C	26.2 ± 0.5^d^	13.3 ± 0.8 ^f^	12.9 ± 0.5^a^	0.97^a^
	N1	29.8 ± 0.7^c^	18.3 ± 0.4^e^	11.5 ± 0.5^b^	0.63^b^
	N2	31.9 ± 0.5^b^	22.3 ± 0.3^d^	9.6 ± 0.4^c^	0.43^c^
	P1	32.3 ± 0.5^b^	22.6 ± 0.6^d^	9.7 ± 0.3^c^	0.43^c^
	P2	32.8 ± 0.4^b^	24.2 ± 0.5^c^	8.6 ± 0.6^d^	0.36^d^
	N1 + P1	33.6 ± 0.6^a^	25.8 ± 0.4^b^	7.8 ± 0.5^e^	0.30^e^
	N2 + P2	34.2 ± 0.6^a^	27.1 ± 0.5^a^	7.1 ± 0.4^e^	0.26^e^

Values in a column with different letters are significantly different from each other according to Tukey’s HSD post hoc test with Bonferroni correction at *P* < 0.01.

The growth of *R. confertus* was stimulated by both N and P fertilization. The plants treated with N1 or P1 fertilizer had significantly (Tukey’s test, *P* < 0.05) greater biomass production (plant dry weight and shoot dry weight) than the control (i.e., 0 g N and P) and the effect was most pronounced in N2 or P2 (i.e., 140 g N and 37.5 g P). In addition, across all soil types, P1 and P2 (i.e., 25 g and 37.5 P) had greater effect than N1 and N2 (i.e., 70 g N and 140 g P). There was also a significant (Tukey’s test, *P* < 0.05) interaction effect of N and P for total biomass, shoot and root biomass and found that values for these parameters were highest in seedlings treated with N2 + P2 (i.e., 140 g N and 37.5 g P). Across all the soil types, the total dry biomass was largest at N2 + P2 combination on Fluvic Cambisol. In contrast to the shoots, growth of the roots appeared to be decreased with increasing amounts of inorganic N and P in the soil.

The R: S ratio was significantly (Tukey’s test, *P* < 0.05) affected by the nutrient treatments. It was highest for *R. confertus* grown on Fluvic Cambisol without N or P addition, whereas the plants growing on Dystric Gleysol with N2 + P2 exhibit the lowest R: S ratio. There was also a N-P interaction effect: R: S ratio tended to be lower when N and P were added in combination (Tukey’s test, *P* < 0.05).

### Leaf N and P concentrations

The leaf concentrations of N and P differed significantly (*P* < 0.01) between plants grown on four soil types and tended to increase with increasing level of soil applied N and P (Table 3). The control plants grown on Fluvic Cambisol without N or P fertilization showed the highest N or P leaf concentrations, significantly exceeding other soils (*p* < 0.001), whereas the lowest N or P leaf concentrations were observed in Dystric Gleysol without N or P. The ranking was: Fluvic Cambisol > Eutric Cambisol > Fibric Histosol > Dystric Gleysol. Fertilization altered leaf N and P concentrations. Plants fertilized with N or P exhibited higher N concentrations than plants without N or P. Across all soil types, N concentrations increased with addition of N alone (Tukey’s test, *P* < 0.001), P alone (Tukey’s test, *P* < 0.05), and N and P together (Tukey’s test, *P* < 0.05). The total foliar P concentration followed a similar trend as the total N with soil type and fertilization affecting leaf P concentration (Table 5). Regardless of the soil type, the highest leaf concentrations of N or P were always noted in the plants fertilized with the highest N or P supply. In all analyzed soils, N: P ratios were below 15.

**Table 3:** The leaf concentrations of nitrogen (N) and phosphorus (P) (mg g^−1^ DW), N: P ratio (expressed as mass ratio gN/gP) of *Rumex confertus* plants fertilized with N and P (individually or in combination) in four types of soil.

Soil Types	Nutrients added	N (mg g^−1^ DW)	P (mg g^−1^ DW)	N: P ratio (g g−1)
Fluvic Cambisol	Control	18.5 ± 1.2	1.47 ± 0.6	12.6
	N1	21.45 ± 0.25	1.68 ± 0.15	12.8
	N2	25.87 ± 0.27	2.08 ± 0.25	12.4
	P1	27.08 ± 0.28	2.15 ± 0.17	12.6
	P2	28.98 ± 0.24	2.32 ± 0.31	12.9
	N1 + P1	32.35 ± 0.41	2.45 ± 0.26	13.3
	N2 + P2	36.22 ± 0.36	2.56 ± 0.46	14.1
Eutric Cambisol	Control	17.6 ± 0.32	1.42 ± 0.14	12.4
	N1	20.36 ± 0.25	1.66 ± 0.11	12.3
	N2	24.12 ± 0.31	1.88 ± 0.34	12.8
	P1	26.06 ± 0.37	1.99 ± 0.36	13.1
	P2	28.34 ± 0.28	2.06 ± 0.42	13.7
	N1 + P1	30.37 ± 0.24	2.15 ± 0.31	14.1
	N2 + P2	32.89 ± 0.36	2.218 ± 0.43	14.4
Fibric Histosol	Control	15.38 ± 0.35	1.32 ± 0.19	11.7
	N1	18.67 ± 0.41	1.53 ± 0.25	12.2
	N2	21.25 ± 0.39	1.65 ± 0.32	12.9
	P1	23.41 ± 0.32	1.85 ± 0.37	12.7
	P2	25.13 ± 0.35	1.97 ± 0.24	12.8
	N1 + P1	27.34 ± 0.41	2.11 ± 0.34	13.0
	N2 + P2	30.90 ± 0.35	2.24 ± 0.23	13.8
Dystric Gleysol	Control	12.67 ± 0.41	1.29 ± 0.25	9.8
	N1	15.25 ± 0.39	1.42 ± 0.22	10.7
	N2	16.41 ± 0.32	1.49 ± 0.27	11.0
	P1	21.32 ± 0.26	1.58 ± 0.29	13.5
	P2	24.13 ± 0.25	1.67 ± 0.24	14.4
	N1 + P1	26.04 ± 0.41	1.81 ± 0.24	14.3
	N2 + P2	28.90 ± 0.35	1.97 ± 0.23	14.7

The values are means ± standard deviations. The values in a column with different letters are significantly different from each other according to Tukey’s HSD post hoc test with Bonferroni correction at *P* < 0.01. N, P, N + P = factorial additions of nitrogen and phosphorus. DW represents dry weight.

### Leaf chlorophyll content

**Figure 1:**
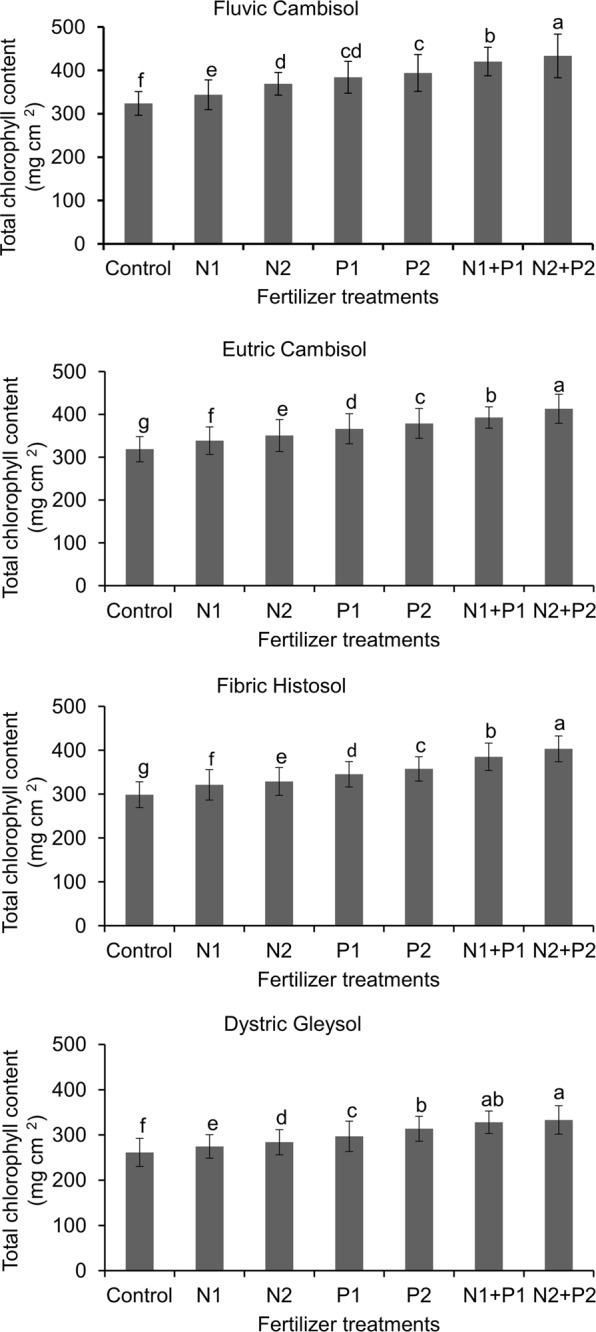
Mean (±SD) leaf chlorophyll content (mg cm^−2^) in *Rumex confertus* plants in relation to the soil types and fertilization treatments. Different letters denote significant differences between mean values (ANOVA, Tukey’s HSD post hoc test with Bonferroni correction at *P* < 0.01). Data log transformer.

Different types of the cultivation soil had significant effects (Tukey’s test, *P* < 0.05) on chlorophyll content in *R. confertus* (Fig. 1) which was the lowest in the plants grown in Dystric Gleysoil. Compared to this in the plants grown in Fluvic Cambisol this parameter was significantly increased by 23% (*P* < 0.01). The supply of both N and P had significant (Tukey’s test, *P* < 0.05) positive effects on the chlorophyll content. The plants treated with N1 had significantly (Tukey’s test, *P* < 0.05) greater levels of chlorophyll than those of the control (0 g N) while in those treated with N2 this parameter was highest. Similarly, the plants treated with P1 or P2 had significantly (Tukey’s test, *P* < 0.05) greater levels of chlorophyll than those treated with 0 g N (control) regardless of the soil type. A significant interaction (*P* < 0.05) effect of N and P on the levels of chlorophyll was also observed, the total chlorophyll was highest in the plants treated with N2 + P2 (Fig. 1).

The three-way ANOVA results revealed that all main factors (soil type ‘S’, nitrogen ‘N’, phosphorus ‘P’) and their interactions had significant effects on morphological and physiological variables (Table 4). The parameters, such as total biomass, root and shoot dry biomass were significantly impacted by all the main effects and interactions; while R: S ratio weakly responded both to single nutrient application and their interactions (Table 6). In addition, the effect of the interaction between nutrient treatments and soil type was found for total chlorophyll content, leaf N and P concentration.

**Table 4:** Results of three-way ANOVA (numbers represent *F*-values) for the effects of the soil type (S), nitrogen (N) and phosphorus (P) on the total dry biomass, shoot dry biomass, root dry biomass, total chlorophyll content and foliar N and P concentrations of *Rumex confertus* seedlings grown with N and P additions (N separated or in combination) in four different natural soils.

Source of variation	Soil type (S)	Nitrogen (N)	Phosphorus (P)	S × N	S × P	N × P	S × N × P
Total dry biomass	34.78***	19.85***	18.85***	3.23*	8.05*	12.89**	18.43*
Shoot dry biomass	25.54***	17.87**	14.78**	5.6*	8.9*	9.8*	6.7*
Root dry biomass	17.56**	18.21**	12.21**	9.8*	12.4*	7.51*	6.0*
R: S ratio	5.32*	6.56*	10.4*	6.56*	8.23*	8.34*	5.46*
Total chlorophyll content	16.31**	18.96**	18.96**	11.78*	18.6*	7.65*	11.07*
Foliar N-concentartion	15.54**	16.56*	10.32*	13.67*	13.22*	16.12*	16.8*
Foliar P-concentration	14.65*	17.34*	16.21*	14.42*	15.51*	10.4*	14.8*

There were four soil types (Fluvic Cambisol, Eutric Cambisol, Fibric Histosol, Gleysol), four nitrogen treatments (N1, N2, N1 + P1, N2 + P2) and four phosphorus treatments (P1, P2, N1P1, N2P2). Each treatment had four replicates. S: soil types, N: nitrogen, P: phosphorus. *P < 0.01, **P < 0.01, ***P < 0.01. S: soil types, N: nitrogen, P: phosphorus. Data log transformer.

**Relative Colimitation Index (RCI**_**N**_**)** (i.e. an **index** of the **relative** limitation of biomass production by **N and P**). In all soils, the mean RCI_N_ index for N was between 0.5 and 0.7, while RCI_P_ for P was between 0.7 and 0.9 (Table 5).

**Table 5:** Relative Colimitation Index (RCI) in relation to soil types and fertilization treatments.

Soil types	RCI_N1_	RCI_N2_	RCI_P1_	RCI_P2_
Fluvic Cambisol	0.6	0.6	0.8	0.8
Eutric Cambisol	0.5	0.5	0.9	0.8
Fibric Histosol	0.6	0.6	0.7	0.7
Gleysols	0.5	0.7	0.8	0.8

In all soils, the mean RCI_N_ index for N was between 0.5 and 0.7, while RCI_P_ for P was between 0.7 and 0.9.

### Rate of emergence

**Figure 2:**
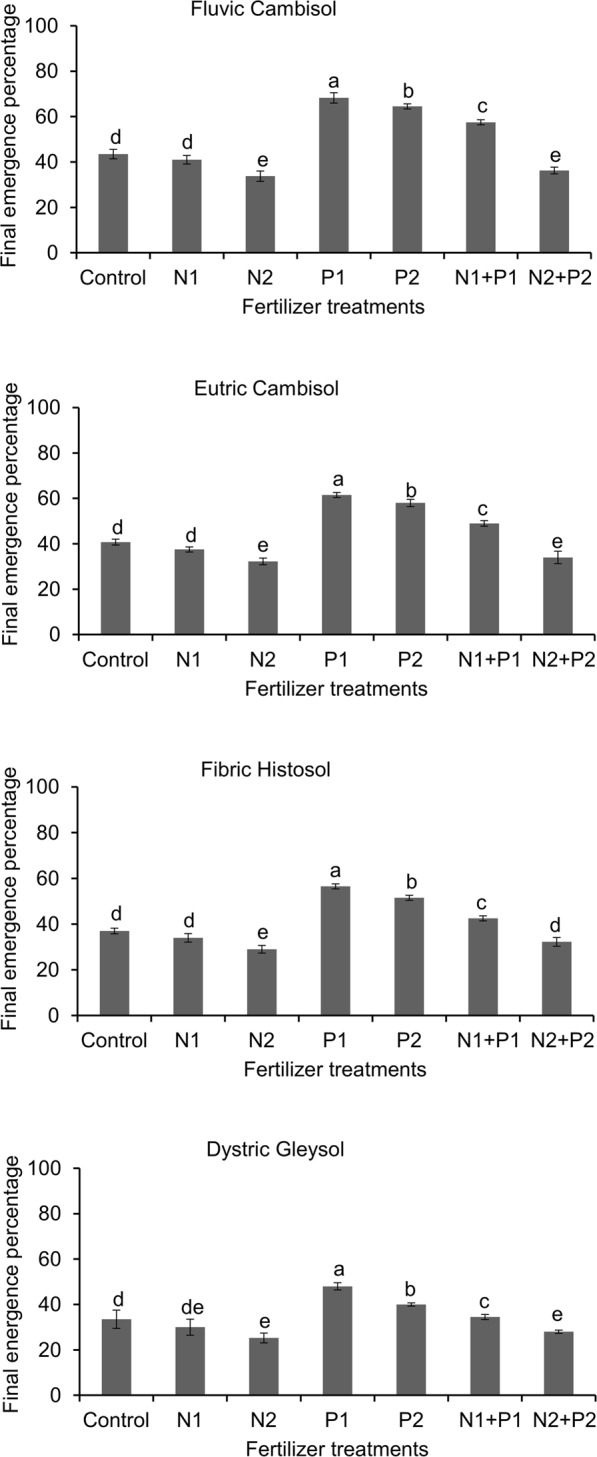
Effects of different fertilizer treatments on field emergence of *Rumex confertus* seedlings grown in four soils. Values in a column with different letters are significantly different from each other according to Tukey’s HSD test with Bonferroni correction at *P* < 0.01). Data log transformer.

The three-way ANOVA indicated that the soil type, nitrogen, phosphorus and their interaction significantly affected final seedling emergence percentage (Table 6). In Fluvic Cambisol, higher emergence values were recorded than in Eutric Cambisol (Fig. 2), but in Eutric Cambisol seedlings were green and substantially more vital than in Fluvic Cambisol. The mean emergence over all levels of nutrient availability was 49, 45, 40 and 38% for Fluvic Cambisol, Eutric Cambisol, Fibric Histosol and Dystric Gleysol, respectively. In all soil types, the emergence was lowest in both treatments with N2 applications (N2 and N2 + P2) and these treatments were significantly different from the P1 and P2 treatments. The highest field emergence, was recorded in P1 followed by P2 treatment.

**Table 6:** Results of three-way ANOVA of characteristics of final seedling emergence of *R. confertus* in relation to soil types and fertilizer treatments.

Independent variables	Final emergence percentage
Soil type (S)	46.37***
Nitrogen (N)	23.57**
Phosphorus (P)	21.24**
S × N	6.45*
S × P	8.46**
N × P	18.41*
S × N × P	14.49*

There were four soil types (Fluvic Cambisol, Eutric Cambisol, Fibric Histosol, Gleysol), four nitrogen treatments (N1, N2, N1P1, N2P2) and four phosphorus treatments (P1, P2, N1 + P1, N2 + P2). Each treatment had four replicates. *Figures represent F-values*. *P < 0.01, **P < 0.01, ***P < 0.001. Data log transformer.

## Discussion

The development and morphology of a particular plant species can by modified by the soil type in which the plant grows. This may explain the significant differences in the growth parameters and nutrient requirements between *R. confertus* plants growing on the tested soil types. For example, the results from experiments 1 indicated that the poor development of *R. confertus* in the Dystric Arenosol, Entic Podzol, Brunic Arenosol and Leptosol was probably a reflection of the low levels of available nitrogen and phosphorus in these soils. Low nutrients availability along with low soil water content are the limiting factors for plant growth on sandy soils i.e., Dystric Arenosl, Entic Podzol and Brunic Arenosol are, thus the species composition of plant communities is determined by competition for nutrients rather than light^24^. Plant species typical of sandy soils have evolved numerous adaptations to the xerothermic microclimatic conditions for example morphological attributes, seed dormancy rhythms and capacity of clonal spread which are not present in *R. confertus*. Although no measurements were taken, visual assessment revealed that the plants grown on sandy soils poor in N showed symptoms typical of this element deficiency^10^; they were small with pale, chlorotic foliage and showed visible darkening of the foliage and pronounced purple discoloration. Typical signs of P deficiency were found, including increased R: S ratio and reduced biomass. Under natural conditions the majority of these plants undoubtedly would die within a short time in such surroundings, and even if a few managed to survive for a longer period probably competition from species of psammophilous grasslands *Spergulo morisonii-Corynephoretum canescentis* (Tx. 1928) Libb. 1933 e.g., *Achillea millefolium* agg., *Corynephorus canescens*, *Helichrysum arenarium, Hieracium pilosella*, *Hypochoeris radicata* and *Jasione montana* would ultimately lead to their death.

Soil variables limiting plant growth and development differ according to the position of the soil in the acidity gradient. Low soil pH-values and low water contents limit plant nutrient uptake^25^. In addition, high H ion activity in these soils elevates concentrations of exchangeable aluminium (Al)^25,26^ or manganese (Mn) ions^27^, which under specific conditions and in appropriate amounts become toxic to plants. It is possible that Al or Mn ions may exclude *R. confertus* from Dystric Arenosol, Entic Podzol and Brunic Arenosol. De Graaf *et al*.^28^ observed that *Arnica montana* and *Cirsium dissectum* showed Al toxicity, indicated by poor root development, yellowish leaves and reduced contents of Mg and P in the plants. However, there are no consistent views about the critical pH value for growth of plants. Several reports indicate maximal Al toxicity at pH around 4.5^28^ but other reports – at pH around 5.5^29^. In acidic soils (soil solution pH < 6.0), iron and aluminum amounts are high, which causes either fixation or removal of P from the soil solution. This limits the availability of inorganic P to plants at soils with pH levels <5.0. Very little is known about germination and seedling establishment on dry acidic soils. It seems that the low water holding capacity of the soil is the main factor limiting germination and seedling survival. Furthermore, mosses such as *Polytrichum piliferum*, which may amount to 70% of total vegetation cover, seem to inhibit germination of herbs and grasses^30^.

The poor development and growth of *R. confertus* on a calcareous Leptosol in the experiment 1 is probably results from the high pH (pH 8–8.5) and exchangeable calcium (Ca). Lime chlorosis in plants grown on calcareous Leptosol was probably attributed to Fe deficiency^31^. In addition, at higher pH levels, calcium may react with phosphorus, creating unavailable phosphate compounds for plants^30^, which may cause poor growth of *R. confertus* on Leptosol. This is consistent with the pattern found in *R. obtusifolius*^32^. High demand for certain trace elements (Fe, Mn, Zn, Cu), which are particularly insoluble at high pH, could also be a possible explanation for the absence of *R. confertus* from calcareous soils^30^.

Different types of cultivation soil in the experiment 2 had different impacts on traits of *R. confertus* (Table 4). For example, both the total plant dry weight and shoot biomass were highest in Fluvic Cambisol, higher by 46% and 60% respectively, as compared to the plants in Dystric Gleysol in which their performances were poorest. The differences in these parameters between two types of soil were statistically significant (*P* < 0.05). The relatively poor growth recorded in Dystric Gleysols could be due to poor root growth, resulting in subsequent reduction in the roots absorptive surface area for both water and nutrients.

The R: S ratio may often provide some estimation of nutrient conditions of soils^33–35^. The mechanisms which regulate nutrient allocation to shoots and roots remain unclear. The nutrient supply hypothesis^36,37^ proposed that plants could increase the relative amount of carbon allocated to the roots (resulting in increased R: S dry weight ratios) under deficiency of N or P but not of K or Mg^38^ or when other environmental factors such as Al toxicity^38^ limit root growth. Consistently with my first hypothesis, it was found that the shoot biomass increased, and that of roots decreased, with increasing N and P availability. Consequently, R: S ratios were lowered by high nutrient availability in all soil types. The R: S ratio for *R. confertus* also decreased as applied P increased consistent with observations on other species, for example for *Trifolium hirtum* and *T. subterraneum*^39^, and *Agave lechuguilla*^40^. Similarly, the R: S ratio decreases as N availability is increased for *Atriplex litoralis*^41^ and *Dactylis glomerata*^42^. However, in some species, such as the perennial leguminous shrub *Lespedeza davurica* and the perennial herbaceous grass *Bothriochloa ischaemum*, P generally stimulates root growth^42^. In other cases, however, not only shoot growth but also root growth of invaders was enhanced by increasing N supply^43^.

Numerous papers reported positive interaction between N and P which led to increase in N or P absorption and higher yields^18,20^. As predicted (hypothesis 2) P fertilization improved leaf nutrient status more than N fertilization, which can explain greater effect of the former on plant growth. In this study across all soil types, N concentrations increased with addition of N alone, P alone, and N and P together. However, N content was higher with P fertilization than with N fertilization.

Foliar N concentrations of *R. confertus* in this experiment were comparable to those measured by Vondráčková *et al*.^44^ in *R. obtusifolius* and *R. acetosa*. Several recent studies showed opposite results, i.e. N concentrations in leaves did not increase with soil N concentration^43^.

Nutrient limitation may be estimated by indirect measurements of some parameters, the most common of which include (1) nutrient availability in soil^45^; (2) plant investments in acquiring particular nutrients^46^; (3) patterns of limitation assessed by analyzing colimitation indices for N and P nutrients^47^, and (4) ratios of nitrogen to phosphorus (N: P) in plant foliage^48–51^. In this study Relative Colimitation Index (RCIN) and the leaf N: P ratio were used as indicators to determine limitation types. It is widely reported that plant growth is N-limited at foliar N: P < 14, P-limited at N: P > 16, and co-limited by N and P at intermediate values^50^. In this study, foliar N: P ratios ranged from 12 to 15, suggesting that growth of *R. confertus* was co-limited by N and P (hypothesis 3). The shoot biomass of *R. confertus* increased with the N and P addition rate, indicating co-limitation by N and P. Growth co-limitation by N and P was also indicated by the increase in N and P concentrations with increasing N and P supplies. Tomassen *et al*.^52^ showed that the N: P ratios could not predict the responses of individual plant species to fertilization because the N: P ratio tool developed by Koerselman & Meuleman^48^ and by Verhoeven *et al*.^15^ is based on the results obtained for the plant community as a whole (i.e. bulk vegetation) rather than for individual species. However, Tessier and Raynal^51^ postulated that N: P ratios may be used to indicate nutrient limitation in individual species. To quantify the relationships between N and P, a Relative Colimitation Index (RCIN) was calculated^46^. The results of this study showed that the mean relative colimitation index for P was between 0.5 and 0.7, and for N between 0.7 and 0.9, suggesting that growth was co-limited by N and P.

If N and P are among the macronutrients considered most limiting to plants growing in grassland soils, plants growing on soils of higher fertility should be less responsive to N and P addition than those growing on soils poor in these macronutrients^53^. My research results support to this hypothesis. In this study, addition of these two macronutrients individually and/or together enhanced biomass production more in poor soils than in soils with high fertility.

Leaf chlorophyll content indicates their photosynthetic capacity because low content of chlorophyll limits the process of photosynthesis and, consequently, leads to a decrease in biomass production in the plants^54^. Chlorophyll synthesis depends upon mineral nutrition^55–58^. In vascular plants, high chlorophyll content is typically correlated with increased N uptake^59,60^, for example, it might be due to the availability of N, which plays role in chlorophyll formation. As predicted (hypothesis 4) total chlorophyll contents significantly increased following fertilization with N and P in different soil type. The effects of nutrient addition on chlorophyll content were in agreement with the previous findings^61,62^.

In this study, seedlings emergence was negatively affected by very high N availability but positively affected by increased P availability in the soil. It was caused by a negative impact of high NH_4_^+^ and/or NO_3_^−^ on seed germination^17^. However, growth tends to be higher with a high N supply. This is consistent with the pattern found in *R. obtusifolius* and *R. crispus*^17,63^.

The results suggest that the poor emergence and subsequent growth of seedlings in Dystric Gleysol was caused by the negative impact of soil compaction. Effect of soil compaction on poor emergence and *R. confertus* growth observed in this study can be due to decreased root growth. Soil compaction affects soil physical characteristics in grasslands^64^. The most obvious physical effect is lack of oxygen^65^, an increase in soil bulk density^66–68^ and the pore spaces reduction^69,70^.

In this study, plants grown in pots filled with soil from the original ecological environment in which they usually grow. However, this study was not designed to reveal the effects of litter traits on germination and seedling emergence. A litter layer sometimes forms a mechanical barrier that prevents seedling emergence and changes environmental factors such as light, soil moisture, and temperature. The effect of litter on seedling emergence and consenquently seedling recruitment in grassland ecosystems could play negative or positive role depending on micro-environmental conditions, thickness of the litter layer and species^71,72^. Therefore, the results of the present study concerning seedlings emergence and growth in pot experiments may therefore have a more general validity despite the use of natural soil.

## Conclusion

To my knowledge, this study evaluated for the first time the interactive effects of soil types and plant nutrition on the growth and seedling emergence of *R. confertus* plants. Based upon these results it is evident that the development and growth of the *R. confertus* were influenced by nitrogen and phosphorus fertilization. Furthermore, P fertilization increased growth more than N fertilization in all four soils used. The concentration and ratio of these fertilizers were important in determining growth and development. Both N and P fertilization affected growth depending on soil properties such as texture and mineral nutrient levels. Regardless of the soil type, the highest N and P concentrations in the leaves were always noted in the plants fertilized with the highest N or P supply.

The results reveal the reasons for the distribution of *R. confertus*. The ability to make use of available nutrients in order to increase biomass and to produce diaspore is one of the reasons for the settlement success of *R. confertus* in grasslands and meadows in the Warta river valley. The nutrient supply to each site can either be related to the factors associated with the river Warta itself (such as its water quality) or with site-specific factors, such as agricultural pollution (inflows of groundwater and surface water). Under these conditions the concentration of nitrogen and phosphorus is sufficiently high to allow *R. confertus* to colonize such sites and become established. Further research is needed to evaluate the effect of grassland management and nutrient availability on plant performance in temperate grasslands and meadows.

## Materials and Methods

### Study species

*Rumex confertus* Willd (Russian or Asiatic dock), probably originating in the Eastern Europe and Asia, where it thrives on meadow-steppes and glades in forest-steppe has been recently observed to successfully colonise Central Europe^73^. The reason for its sudden increase in population growth is not known, although an extended period of germination, rapid growth and high production of small seeds, regeneration of plants form fragments of underground organs, quick and large biomass production and lack of native herbivores were suggested to facilitate its success and spread in Europe^74^. In Poland, where it is an exotic plant, *R. confertus* is considered an aggressive colonizer of meadows, wet ditches, riparian-scrub, roadsides, railway tracks and embankments. Under nutrient-rich soil conditions, *R. confertus* is a very strong competitor^75^. *R. confertus* occurs over a wide range of soil types in Poland, whereas it typically grows on moist soils in its native habitat (personal observations). *Rumex confertus* is a perennial herb (Polygonaceae) with stems of 60–120 cm height. It flowers in July and produces one or usually more inflorescences that can reach up to 0.3 m or more. The seeds of *R. confertus* undergo a seasonal dormancy cycle with deep dormancy in winter and early spring and a low level of dormancy in early autumn^75^.

### Experimental design and growth conditions

Mature *R. confertus* seeds were collected (September 2017) from twenty plants selected randomly, taking care not to favor tall or small, growing in the valley of the Warta River (60 km west of Lodz; N 51°96′, E18°79′; central Poland); the seeds were mixed to obtain one representative seed sample. The collection site was a high productive wet meadow on Eutric Cambisol soil. Subsequently, the seeds were stored at room temperature, in paper bags in the dark for two weeks, Deformed and damaged seeds were discarded, and healthy seeds were exposed to cold stratification (±5 °C in darkness) until use. Prior to sowing the fertilizers were added and thoroughly mixed into the soil. Up to this time, fertilizers were not used routinely. Three experiments were conducted during 2018 in a greenhouse conditions under natural illuminations with a 12–15-h photoperiod (no extra illumination). The temperature by day was 20–30 °C, night 15–20 °C and relative humidity of 30–50%. The objectives, growth conditions, soil treatments, and statistical analyses for each experiment are described below. Germination of the seeds tested under laboratory conditions in a 12-h day/night light at 22 °C directly before establishment of the experiment was 60–70%. In both experiment 1 and 2, I used 5 L polythene pots 25 cm in diameter and 22 cm deep (491 cm^2^) filled (leaving 1 cm brim at the top) with air dried soil (approximately, 7 kg of soil). Subsequently, ten seeds of *R. confertus* were sown (1–2 cm deep) in each pot and after 3 weeks of growth, the seedlings were thinned to one plant in each pot. The seedlings were irrigated with tap water daily. Every week, the pots were reshuffled to avoid light/shade or any other green house effects. Nutrient treatments were initiated 2 days after planting. There were four replicate plants for all treatments in all experiments. Germination succeeded well (70–80%) with all soils and was completed within 14 d. The pots were covered with aluminium foil to prevent evaporation. At the end of an experiment the plants were harvested and washed in deionised water for a short time.

### Soil sampling

The soils for the experiments were collected from eight selected locations, situated in central Poland, which represent eight different habitats (Table 7). All soils were collected from fallows under multispecies plant community that was devoid of alien plants. Soil samples were taken from the top 20 cm of each soil and then was used as the growth substrate. At each site, 4-m^2^ (2 × 2 m) plot with homogeneous soil was designated. In each plot five soil cores with a length of 20 cm and a diameter of 3 cm were collected with a soil borer (one core at each corner of the square and one core at the centre of the square). These five cores were mixed up into a single bulk sample for each plot.

**Table 7:** Basic characterization of sampling locations. ^a^The name given in bracket indicates physico-geographical mesoregion of Poland^80^.

Soil types according IUSS Working Group WRB^81^	Locations^a^	Habitat and land use
Dystric Arenosol	Poddębice (Wysoczyzna Łaska)	Poor acidic grassland with *Corynephorus canescens*, *Helichrysum arenarium*, *Rumex acetosella* and *Potentilla argentea*
Entic Podzol	Zakrzew (Wysoczyzna Łaska)	A community with *Pinus sylvestris*, *Betula verrucosa* and *Juniperus communis*
Brunic Arenosol	Zakrzew (Wysoczyzna Łaska)	Acidophilous oak forest (Quercus robur)
Leptosol	Stanisławów (Kotlina Kolska)	Poor calcareous grassland
Fibric Histosol	Niewiesz-UŁany (Wysoczyzna Łaska)	Wet grassland with *Deschampsia flexuosa* and *Molinia caerulea*
Fluvic Cambisol	Uniejów (Kotlina Sieradzka)	Riverbank with *Phalaris arundinacea* and *Urtica dioica*
Dystric Gleysols	Niewiesz (Wysoczyzna Łaska)	Unmanaged grassland with *Holcus lanatus* and *Agrostis alba*
Eutric Cambisol	Uniejów (Kotlina Sieradzka)	Unmanaged meadow with *Dactylis glomerata* and *Arrhenatherum elatius* outside river valley

### Soil substrate analysis

The soil samples were air-dried at 25 °C for 3 days, after removal of roots, they were ground and passed through a 2-mm sieve to remove rocks, then thoroughly mixed. Next the soils were analyzed by the Laboratory of Chemical-Agricultural Station in Lodz. Soil composites were analyzed for texture, pH, carbon (C), available P, potassium (K), total N and carbon/nitrogen (C: N ratio). The soil particle size distributions of each sample was determined using the laser diffraction method^76^: clay (<0.002 mm), silt (0.002–0.05 mm), sand (0.05–2.0 mm) and soil skeleton particles (>2.0 mm). Organic matter contents of the soil, were estimated as loss on ignition (550 °C, 3 h). Soil pH was determined by mixing a slurry of ten g of air-dried soil with 5 mL deionized water (pH_H2O_), and also after adding 5 drops of 1 M KCl solution to the slurry (pH_KCl_). The Tiurin method was applied to assess the total carbon content. Total N was extracted using micro-Kjeldahl method based on the wet oxidation of organic matter using 30 M sulfuric acid (H_2_SO_4_), and then, the concentration was determined using the indophenol blue method. The contents of phosphorus and potassium were determined based on the Egner-Riehm method^77^.

Total N and P content in leaf tissue was determined by acid digestion (340 °C) of ground (to fine powder using a ball mill) after dried (60 °C for 72 h) plant material (subsamples of 100 mg) with a mixture of salicylic acid and sulphuric acid using a selenium mixture’as catalyst (a modification of the Kjeldahl method). Concentrations in the digests were determined colorimetrically using the molybdenum blue method for phosphorus and the indophenol blue method for nitrogen.

### Experiment 1

To gain some indication of the tolerance of plants to the soil type they were grown from seeds in soils which do not support this species under natural conditions. Establishment of *R. confertus* form seeds was studied in a greenhouse experiments using four contrasted soil types: Dystric Arenosol, Entic Podzol, Brunic Arenosol and Leptosol. The duration was 10 weeks: sowing date 8 May, harvest date 18 July, 2018. Subsequently, ten seeds of *R. confertus* were sown (1–2 cm deep) in each pot and after 3 weeks of growth, the seedlings were thinned to one plant in each pot. A total number of 12 pots were randomly divided into for groups for soil type.

### Experiment 2

This was a two-factor experiment involving soil type and nutrient treatments. A fertiliser experiment was carried out to determine whether nitrogen or phosphorus was limiting plant growth at four soil types which support this species under natural conditions. Based on the characteristics of the natural distribution of *R. confertus*, I selected four representative soil types (i.e., Calcaric Leptosol, Fibric Histosol, Fluvic Cambisol and Eutric Cambisol) from four different sites with quite different textures and physicochemical properties. The sites fulfilled the following conditions: (i) having well established and still expanding populations of *R. confertus*; (ii) having sufficiently homogenous soil; (iii) the survey should include a range of habitats from nutrient poor grasslands to highly productive meadows, and (iv) little or no active grazing or herbicide use. At each site where this invader species was found, one plot was marked out (from which soil samples were taken) nearby the areas that had no evidence of invasion.

From the beginning of May 2018 until the end of September 2018 the plants were grown under glasshouse conditions. The seeds were sown as in experiment 1. At sowing time (10 Mai) the dry, granular fertilizers were added and thoroughly mixed into the soil. Seven nutrient treatments i.e., single-nutrient (N and P) and dual-nutrient (N + P, herein NP) were used in this research, differing in the amount of N or P: N1, N2, P1, P2, N1 + P1, N2 + P2, and a control without any fertilized input (Table 8). Nitrogen was applied as ammonium nitrate with lime (NH_4_NO_3_ + CaCO_3_, containing 27.5% N, 10% Ca) ─ N treatment and P was applied as super phosphate (Ca(H_2_PO_4_)_2_ + CaSO_4_, 8.5% P, 20% Ca, 10% S) ─ P treatment. There were also combined N and P and control (only demineralised water) treatments as indicated in Table 1. Fertilizer rates were calculated on a surface area basis. The design was a 4 × 7 factorial (soil type × N–P treatments) for a total of 28 replicated four times and arranged in a randomized complete block, i.e. 112 pots per experiment with each pot being considered an experimental unit.

**Table 8:** Fertilizer treatments and the amount of nutrients application. ^a^Number in bracket is equivalent to the total amount in kg ha^−1^ of the applied nutrients.

Treatment	Nutrients added (mg pot^─1^ year^−1^)	
	N	P
Control	—	—
N1	70 (^a^150)	—
N2	140 (300)	—
P1	—	25 (50)
P2	—	37.5 (75)
N1 + P1	70 (150)	25 (50)
N2 + P2	140 (300)	37.5 (75)

The plants were harvested after a growth period of five months and their biomass divided into roots and shoots (i.e., stems with leaves). The roots were first washed thoroughly with tap water to remove soil adhered to belowground organs. The total plant biomass (g plant^−1^) was the sum of root, stem and leaf biomass. Dry weight of the plant parts was measured after drying the material for at least 72 h at 60 °C. Dry biomass was obtained by weighing individual floral parts in a microbalance. From these primary data the root to shoot (R: S) ratio was calculated as the root dry biomass divided by shoot biomass. To collect and prepare leaf samples the first mature leaf (blade plus petiole) was taken from each plant per treatment combination in each soil type. After drying, the leaves were ground with a miniature coffee mill to pass through a 0.7-mm mesh screen. The leaf samples were then analyzed for N and P concentrations (% dry mass), as in the soil samples.

Relative Colimitation Index (RCI_N_)^47^ was used to quantify the relationships between N and P. The equation was as follows:

1 $${{\rm{RCI}}}_{{\rm{N}}}=({{\rm{B}}}_{{\rm{N}}}-{{\rm{B}}}_{{\rm{C}}})/({{\rm{B}}}_{{\rm{N}}+{\rm{P}}}-{{\rm{B}}}_{{\rm{C}}})$$

where, Bc is the biomass of unfertilized plants (control), B_N_ is the biomass of plants fertilized with N, and B_N+P_ is the biomass of plants fertilized with N and P. RCI_N_ value between 0 and 0.5 indicates that the plants are more classically co-limited by N and P than they are primarily limited by N. If RCI_N_ is between 0.5 and 1, plants are primarily limited by N. By analogy to N, an index was also calculated for P.

Leaf chlorophyll content was determined by measuring the ratio between the chlorophyll fluorescence at 735 nm and 700–710 nm^78^ by a hand-held instrument (CCM300, Opti-Sciences, Inc., Hudson, USA), using standard manufacturer-recommended protocols. The working principal and design of CCM-300 is based on the work of Gitelson *et al*.^76^ using the equation (Chl) = 634 × F735/F700 + 391. The chlorophyll content (mg/m^2^) of the same plant in each treatment was measured in recently fully expanded leaf (from the tip, center, and base of each leaf) per each plant. Mean values of the three sections were used for data analysis.

### Experiment 3

I conducted a glasshouse experiment to study the effects of N and P addition and soil type on seedlings emergence. All materials and procedures for this experiment were as for experiment 2, except as noted below. In a previous paper^74^, it was shown that sand burial depth had significant effect on *R. confertus* seedling emergence. Only from the seeds buried at the depth of 0.5 cm, 15% seedling emergence was observed. At the depths >0.5 cm, the percentage of seedling emergence was almost zero. Therefore, in this experiment, the seeds were sown at the depth of 0.5 cm.

The seeds were sown into celled-flats, each of which consistent of 30 square cells (2.5 × 2.5 × 5 cm). The sowing density was 1 seed per cell. Thirty cells were sown for each soil type and subsequently thirty seedlings were treated separately as replication in each case. The design was a 4 × 7 factorial (soil type × fertilizer treatment) replicated four times and arranged in a randomized complete block, i.e. 28 flats or 840 cells were sown in total with each flat being considered an experimental unit. The flats were watered if necessary to maintain optimal growth conditions. The duration was three weeks; sowing date was 7 July and the cumulative number of seedlings (field emergence) was recorded up to 31 July as no further increase in number of seedlings was recorded after this date. Emergence was defined as the protrusion of cotyledons above the soil surface.

### Statistical analysis of the data

Analyses of variance (ANOVA) were performed for total plant biomass, Chl content, leaf tissue mineral element concentrations as well as R: S and N: P values. Tukey’s post-hoc test was used to identify differences between treatments. Differences were considered significant at a probability of *P* < 0.05. The effects of soil types and different N and P concentrations on morphological and physiological variables were analyzed by three-way factorial ANOVA. Normality was verified with the Shapiro-Wilk test. Prior to statistical analysis, the data were log-transformed to make the variance less dependent on the means and to fit a normal distribution. Statistical analyses were performed using the Statistica 13.0 package^79^.

## Data Availability

All data generated or analyzed during this study are included in this published article.

## References

[1] Pyšek, P. & Richardson, D. M. Traits associated with invasiveness in alien plants: Where do we stand? in Biological invasions, Ecological Studies 193 (ed Nentwig, W.) 97–126 (Springer Verlag, Berlin & Heidelberg 2007).

[2] Hejda M, Pyšek P, Jarošik V (2009). Impact of invasive plants on the species richness, diversity and composition of invaded communities. J. Ecol..

[3] Haukka AK, Dreyer LL, Esler KJ (2013). Effect of soil type and climatic conditions on the growth and flowering phenology of three Oxalis species in the Western Cape, South Africa. S. Afr. J. Bot..

[4] Quan M, Liang J (2017). The influences of four types of soil on the growth, physiological and biochemical characteristics of Lycoris aurea (L’ Her) Herb. Sci. Rep..

[5] Prinzing A, Durka W, Klotz S, Brandl R (2002). Which species become aliens?. Evol. Ecol. Res..

[6] Williamson MH, Fitter A (1996). The characters of successful invaders. Biol. Conserv..

[7] González AL (2010). Can ecological stoichiometry help explain patterns of biological invasions?. Oikos.

[8] Chapin FS (1980). The mineral nutrition of wild plants. Annu. Rev. Ecol. Syst..

[9] Shaver GR, Chapin FS (1995). Long-term responses to factorial NPK fertilizer treatment by Alaskan wet and moist tundra sedge species. Ecography.

[10] Marschner, H. Mineral nutrition of higher plants. (Academic Press, London 1995).

[11] Elser JJ (2003). Growth rate-stoichiometry couplings in diverse biota. Ecol. Lett..

[12] Elser JJ (2007). Global analysis of nitrogen and phosphorus limitation of primary producers in freshwater, marine and terrestrial ecosystems. Ecol. Lett..

[13] Brooks ML (2003). Effects of increased soil nitrogen on the dominance of alien annual plants in the Mojave Desert. J. Appl. Ecol..

[14] Zedler JB, Kercher S (2004). Causes and consequences of invasive plants in wetlands: opportunities, opportunists, and outcomes. Crit. Rev. Plant Sci..

[15] Thorpe AS, Archer V, DeLuca TH (2006). The invasive forb Centaurea maculosa increases phosphorus availability in Montana grasslands. Appl. Soil Ecol..

[16] Verhoeven JTA, Schmitz MB (1991). Control of plant growth by nitrogen and phosphorus in mesotrophic fens. Biogeochemistry.

[17] Křišťálová V, Hejcman M, Červená K, Pavlů V (2011). Effect of nitrogen and phosphorus availability on the emergence, growth and over-wintering of Rumex crispus and Rumex obtusifolius. Grass Forage Sci..

[18] Adams, F. Interactions of Phosphorus with Other Elements in Soil and Plants in The Role of Phosphorus in Agriculture (ed. Dinauer, R. C.) 655–680 (American Society of Agronomy, Madison, WI 1980).

[19] Harpole S (2011). Nutrient co-limitation of primary producer communities. Ecol. Lett..

[20] Graciano C, Guiamet JJ, Goya JF (2006). Fertilization with phosphorus increases soil nitrogen absorption in young plants of Eucalyptus grandis. Forest Ecol. Manage..

[21] Sumner ME, Farina MPW (1986). Phosphorus interactions with other nutrients and lime in field cropping systems. Adv. Soil Sci..

[22] Fageria, N. K., Baligar, V. C. & Jones, C. A. Growth and Mineral Nutrition of Crop Plants. (Marcel Dekker, Inc., New York 1997).

[23] Wilkinson, S. R., Grunes, D. L. & Sumner, M. E. Nutrint Interactions in Soil and Plant Nutrition in Handbook of Soil Science (ed Sumner, M. E.) 89–112 (CRC Press, Boca Raton, FL 1999).

[24] Tilman D (1997). Community invasibility, recruitment limitation, and grassland diversity. Ecology.

[25] Tyler G (1992). Inability to solubilize phosphate in limestone soils–key factor controlling calcifuge habit of plants. Plant Soil.

[26] Roem WJ, Klees H, Berendse F (2002). Effects of nutrient addition and acidification on plantr species diversity and seed germination in heathland. J. Appl. Ecol..

[27] Walderen S, Davies MS, Etherington JR (1987). The effect of manganese on root extension of Geum rivale L., G. urbanum L. and their hybrids. New Phytol..

[28] De Graaf MCC, Bobbink R, Verbeek PJM, Roelofs JGM (1997). Aluminium toxicity and tolerance in three heathland species. Water Air Soil Poll..

[29] Ryan PR, Delhaize E (2010). The convergent evolution of aluminium resistance in plants exploits a convenient currency. Funct. Plant Biol..

[30] Jeschke M, Kiehl K (2008). Effects of a dense moss layer on germination and establishment of vascular plants in newly created calcareous grasslands. Flora.

[31] Kolesch H, Oktay M, Höfner W (1984). Effect of iron chlorosis-inducing factors on the pH of the cytoplasm of sunflower (Helianthus annuus). Plant Soil.

[32] Humphreys J, Jansen T, Culleton N, Macnaeidhe FS, Storey T (1999). Soil potassium supply and Rumex obtusifolius and Rumex crispus abundance in silage and grazed grassland swards. Weed Res..

[33] Bloom AJ, Chapin FS, Mooney HA (1985). Resource limitation in plants - an economic analogy. Annual Rev. Ecol. Syst..

[34] Andrews M (1993). Nitrogen effects on the partitioning of dry matter between shoot and root of higher plants. Curr. Top. Pl. Physiol..

[35] Andrews M, Sprent JI, Raven JA, Eady PE (1999). Relationships between shoot to root ratio, growth and leaf soluble protein concentration of Pisum sativum, Phaseolus vulgaris and Triticum aestivum under different nutrient deficiencies. Plant Cell Environ..

[36] Brower R (1962). Nutrient influences on the distribution of the dry matter in the plant. Nhetherlands J. Agric. Sci..

[37] Hermans C, Hammond JP, White PJ, Verbruggen N (2006). How do plants respond to nutrient shortage by biomass allocation?. Trends Plant Sci..

[38] Ericsson T (1995). Growth and shoot: root ratio of seedlings in relation to nutrient availability. Plant Soil.

[39] Raguse CA, Taggard KL (1979). Growth of subterranean clover in a range soil as affected by microclimate and phosphorus availability. III. Comparative growth of subterreanean and rose clovers at cold soil temperatures. Agron. J..

[40] Nobel PS, Quero E, Linares H (1989). Root Versus Shoot Biomass: Responses to Water, Nitrogen, and Phosphorus Applications for Agave lechuguilla. Bot. Gaz..

[41] Steen E (1984). Root and shoot growth of Atriplex littoralis in relation to nitrogen supply. Oikos.

[42] Caloin MA, Khodre E, Atry M (1980). Effect of nitrate concentration on the root:shoot ratio in Dactylis glomerata L. and the kinetics of growth in the vegetative phase. Ann. Bot..

[43] Liu G, Yang YB, Zhu ZH (2018). Elevated nitrogen allows the weak invasive plant Galinsoga quadriradiata to become more vigorous with respect to inter-specific competition. Sci. Rep..

[44] Vondráčková S, Hejcman M, Száková J, Müllerová V, Tlustoš P (2014). Soil chemical properties affect the concentration of elements (N, P, K, Ca, Mg, As, Cd, Cr, Cu, Fe, Mn, Ni, Pb, and Zn) and their distribution between organs of Rumex obtusifolius. Plant Soil.

[45] Powers RF (1980). Mineralizable soil nitrogen as an index of nitrogen availability to forest trees. Soil Sci. Soc. Am. J..

[46] Harrison AF, Helliwell DR (1979). A bioassay for comparing phosphorus availability in soils. J. Appl. Ecol..

[47] Craine JM, Jackson RD (2010). Plant nitrogen and phosphorus limitation in 98 North American grassland soils. Plant Soil.

[48] Koerselman W, Meuleman AFM (1996). The vegetation N: P ratio: a new tool to detect the nature of nutrient limitation. J. Appl. Ecol..

[49] Aerts R, Chapin FS (2000). The mineral nutrition of wild plants revisited: a re-evaluation of processes and patterns. Adv. Ecol. Res..

[50] Güsewell SN (2004). P ratios in terrestrial plants: variation and functional significance. New Phyt..

[51] Tessier JT, Raynal DJ (2003). Use of nitrogen to phosphorus ratios in plant tissue as an indicator of nutrient limitation and nitrogen saturation. J. Appl. Ecol..

[52] Tomassen HBM (2004). Expansion of invasiwe species on ombrotrophic bogs: desiccation on high N deposition?. J. Appl. Ecol..

[53] Chapin FS, Vitousek PM, Van Cleve K (1986). The nature of nutrient limitation in plant communities. Am. Nat..

[54] Naumann JC, Young DR, Anderson JE (2008). Leaf chlorophyll fluorescence, reflectance, and physiological response to freshwater and saltwater flooding in the evergreen shrub, Myrica cerifera. Environ. Exp. Bot..

[55] Jose S, Merritt S, Ramsey CL (2003). Growth, nutrition, photosynthesis and transpiration responses of long leaf pine seedlings to light, water and nitrogen. Forest Ecol. Manag..

[56] Bown HE, Watt MS, Clinton PW, Mason EG (2010). Influence of ammonium and nitrate supply on growth, dry matter partitioning, N uptake and photosynthetic capacity of Pinusradiata seedlings. Trees.

[57] Song CJ (2010). Interactive effects of water, nitrogen and phosphorus on the growth, biomass partitioning and water-use efficiency of Bauhinia faberi seedlings. J. Arid Environ..

[58] Waraich EA, Ahmad Z, Ahmad R, Saifullah, Ashraf MY (2015). Foliar applied phosphorous enhanced growth, chlorophyll contents, gas exchange attributes and PUE in wheat (Triticum aestivum L.). J. Plant Nutr..

[59] Evans JR (1989). Photosynthesis and nitrogen relationships in leaves of C3 plants. Oecologia.

[60] Minotta G, Pinzauti S (1996). Effects of light and soil fertility on growth, leaf chlorophyll content and nutrient use efficiency of beech (Fagus sylvatica L.) seedlings. Forest Ecol. Manage..

[61] Shaw B, Thomas TH, Cooke DT (2002). Responses of sugar beet (Beta vulgaris L.) to drought and nutrient deficiency stress. Plant Growth Regul..

[62] Van den Berg AK, Perkins TD (2004). Evaluation of portable chlorophyll meter to estimate chlorophyll and nitrogen contents in sugar maple (Acer saccharum Marsh.) leaves. Forest Ecol. Manage..

[63] Hejcman M (2012). Effect of quick lime and superphosphate additives on emergence and survival of Rumex obtusifolius seedlings in acid and alkaline soils contaminated by As, Cd, Pb, and Zn. Plant Soil Environ..

[64] Beckett CTS (2017). Compaction conditions greatly affect growth during early plant establishment. Ecol. Eng..

[65] Schumacher TE, Smucker AJM (1984). Effect of soil compaction on root growth and uptake of phosphorus. Plant Soil.

[66] Kooistra MJ, Schoonderbeek D, Boone FR, Veen BW, van Noordwijk M (1992). Root-soil contact of maize, as measured by a thin-section technique. II. Effects of soil compaction. Plant Soil.

[67] Cambi M, Certini G, Neri F, Marchi E (2015). The impact of heavy traffic on forest soils: a review. Forest Ecol. Manage..

[68] Henderson CWL (1989). Using a penetrometer to predict the effects of soil compaction on the growth and yield of wheat on uniform, sandy soils. Aust. J. Agr. Res..

[69] Smucker AJM, Erickson AE (1987). Anaerobic stimulation of root exudates and diseases of peas. Plant Soil.

[70] Chen YL (2014). Root architecture alteration of narrow-leafed lupin and wheat in response to soil compaction. Field Crop Res..

[71] Carson WP, Peterson CJ (1990). The role of litter in an old field community: impact of litter quality in different seasons on plant species richness and abundance. Oecologia.

[72] Foster BL, Goss KL (1998). Species richness in a successional grassland: effects of nitrogen enrichment and plant litter. Ecology.

[73] Almazova DI, Rabornov TA (1953). K. biologii shchavelya konskogo (Rumex confertus Willd.). I. Semennoe razmnozhenie shchavelya konskogo (Rumex confertus Willd.) (I. Generative reproduction of the Russian Dock). Biulletin Moskovskogo Obschchestva Ispyttatelei Prirody Otdel Biologicheskii.

[74] Kołodziejek J, Patykowski J (2015). Effect of environmental factors on germination and emergence of invasive Rumex confertus in central Europe. Sci. World J..

[75] Kołodziejek J (2019). Growth and competitive interaction between seedlings of an invasive Rumex confertus and of co-occurring two native Rumex species in relation to nutrient availability. Sci. Rep..

[76] Gitelson AA, Peng Y, Masek JG (2012). Remote estimation of crop gross primary production with Landsat data. Remote Sens. Environ..

[77] Stat-Soft Inc. Statistica for Windows. (Stat-soft Inc., Tulsa, 2016).

[78] Solon J (2018). Physico-geographical mesoregions of Poland: verification and adjustment of boundaries on the basis of 524 contemporary spatial data. Geogr. Pol..

[79] IUSS Working Group WRB. World Reference Base for Soil Resources 2014, update 2015 International soil classification system for naming soils and creating legends for soil maps. (World Soil Resources Reports No. 106. FAO, Rome, Italy 2015).

[80] Arriaga FJ, Lowery B, Mays MD (2006). A fast method for determining soil particle size distribution using a laser instrument. Soil Sci..

[81] Ostrowska, A., Gawliński, S. & Szczubiałka, Z. Metody analizy i oceny właściwości gleb i roślin. (Wyd. Instytut Ochrony Środowiska, Warszawa (1991).

